# Computationally efficient x‐ray simulation framework using parameterized material attenuation models in anatomically detailed imaging

**DOI:** 10.1002/mp.70255

**Published:** 2026-01-08

**Authors:** Martina Nassi, Mikhail Mikerov, Koen Michielsen, Ioannis Sechopoulos

**Affiliations:** ^1^ Department of Medical Imaging Radboud University Medical Center Nijmegen the Netherlands; ^2^ Dutch Expert Centre for Screening (LRCB) Nijmegen the Netherlands; ^3^ Technical Medical Centre University of Twente Enschede the Netherlands

**Keywords:** anatomical realism, computed tomography, image simulation, parameterization model, spectral imaging, tomosynthesis, virtual clinical trials, x‐ray imaging

## Abstract

**Background:**

Virtual clinical trials provide an efficient alternative to clinical imaging trials for evaluating imaging technologies. In x‐ray simulations, however, modeling material‐specific attenuation becomes computationally intensive as anatomical complexity and material heterogeneity in digital phantoms increase. Parameterization models offer a potential solution by representing material properties with a compact set of coefficients.

**Purpose:**

To develop and validate an x‐ray simulation framework that models material attenuation using parameterization models, reducing computational cost while maintaining accuracy.

**Methods:**

Material attenuation was modeled with a five‐coefficient parameterization derived from physical cross‐section data. Unlike conventional ray‐tracing, which projects each material separately, the proposed method projects only the five parameter maps, making computational cost independent of phantom complexity. This framework was evaluated in two scenarios: breast imaging with 10 compressed breast phantoms with varying fibro‐glandular content, and whole‐body imaging with head and abdomen phantoms. Accuracy was assessed by computing percent errors in attenuation coefficients, sinograms, and reconstructed images relative to the conventional approach. For whole‐body imaging only, additional analyses included the impact of resolution loss and noise, the comparison with errors introduced by different projector models to place results in the context of standard simulation variability, and computational time measurements.

**Results:**

Across all materials and both applications, the maximum attenuation coefficient error was 0.007% (breast skin tissue), far below reported biological variability. Projection and reconstruction errors remained within ± 0.006% for all cases. In whole‐body imaging, these errors were well below those from projector model differences (± 0.5%), and image modification routines further concentrated the error distribution around zero. Simulation times decreased significantly, with acceleration factors scaling linearly with the number of materials within the phantoms.

**Conclusions:**

The proposed framework achieves accurate and efficient simulation of material attenuation in x‐ray imaging, especially in anatomically complex scenarios. Validated in both breast and whole‐body imaging, it offers a robust and efficient alternative to conventional methods, supporting the development of advanced virtual clinical trials and spectral imaging research.

## 1 INTRODUCTION

The accelerating advancement of medical imaging technologies poses challenges to comprehensively evaluating and optimizing their design and clinical use. While clinical imaging trials are the gold standard for evaluation, they are often impractical due to ethical limitations, high costs, time requirements, or lack of ground truth. Virtual clinical trials offer a practical alternative by simulating patient anatomy, imaging systems, and interpreters to enable efficient and scalable assessment in a controlled environment.^1^, ^2^

At present, deterministic and Monte Carlo (MC) methods are the two main simulation approaches used in this context. Deterministic techniques offer computational efficiency but rely on simplifying assumptions that may not model stochastic phenomena accurately. MC simulations, on the other hand, use probabilistic modeling to accurately simulate physical phenomena, though at the cost of significant computational demand.^3^, ^4^

In deterministic simulations, which are the focus of this work, each phantom voxel is assigned specific attenuation properties based on material type and composition, and x‐ray projections are computed by integrating the contributions of each material along the x‐ray path. However, this process becomes increasingly burdensome as the number of materials grows, with modern eXtended Cardiac‐Torso (XCAT) phantoms including up to 140 distinct structures,^5^ and as anatomical realism increases with the possible inclusion of inter‐ and intra‐patient material variability.^6^ The need to model materials and their compositional variability accurately and realistically, while maintaining computational efficiency, remains a challenge.

To address this, parameterization strategies offer a promising solution by expressing material attenuation properties using a compact set of coefficients. Early parameterization approaches include basis‐material decomposition models, which represent materials as combinations of two or more standard materials, for example, bone/water or aluminum/polymethyl methacrylate (PMMA), and empirical models based on the Bragg–Pierce power law^7^ and Klein–Nishina cross‐section,^8^ which express attenuation as a function of atomic number and photon energy. The former models are commonly used in material decomposition for spectral computed tomography (CT), but they can introduce systematic errors when the sample composition cannot be fully represented by the chosen basis materials and may lead to inverse crimes if the same basis materials are used for both simulation and system optimization.^9^ An inverse crime occurs when the same model is used to synthesize, as well as to invert, data in an inverse problem, leading to unrealistically accurate results.^10^, ^11^ The latter are generally accurate only over narrow energy ranges. More advanced Taylor‐expansion–based parameterizations^12^, ^13^ improve accuracy for elemental cross‐sections but are limited in recovering compositional information.^12^ In contrast, Midgley's parameterization^14^ provides a generalizable, physically consistent model for arbitrary mixtures across a wide diagnostic energy range, using a finite set of coefficients derived from physical cross‐section data. This makes it suitable for efficient and accurate modeling of complex or heterogeneous phantoms without overlapping with spectral decomposition models.

In this study, we present a simulation framework built on Midgley's parameterization, and we demonstrate its potential through two use cases: simulating fibro‐glandular tissue variability in digital breast tomosynthesis (DBT) and modeling different materials in whole‐body CT.

## 2 MATERIALS AND METHODS

This section first outlines the material parameterization method and its integration into the simulation pipeline, then demonstrates its application in the two proposed case studies.

### 2.1 Material parameterization

The parameterization scheme proposed by Midgley,^14^ based on Benoist's^15^ empirical model and the Jackson‐Hawkes^12^ parameterization, represents the x‐ray linear attenuation coefficient of any material as a linear combination of *N* orthogonal coefficients:

(1) $$\mu \left(Z_{mix},E\right)=\sum_{k=1}^{N}S\left(E,k\right)\;M\left(Z_{mix},k\right).$$

where S(E,k) are energy‐dependent coefficients and $M(Z_{mix},k)$ are composition‐dependent parameters. The mixture parameters are defined as:

(2) $$M\left(Z_{mix},k\right)=\sum_{Z}n\left(Z\right)Z^{k}\;,$$

where n(Z) is the particle density of the element Z. The first mixture parameter represents electron density $N_{e}$, while the remaining parameters are higher‐order statistical moments describing the elemental composition per unit volume. To decouple the effects of particle density from those of sample composition, compositional ratios are introduced:

(3) $$R\;\left(Z_{mix},k\right)={\left(\frac{M\left(Z_{mix},k\right)}{N_{e}}\right)}^{\frac{1}{k-1}}.$$

These ratios have the same dimensionality as atomic number, are always positive, and are equal for pure elements. For mixtures, the ratios satisfy the ordering:

(4) $$Z_{min}\leq R\left(Z_{mix},2\right)\leq \text{…}\leq R\left(Z_{mix},N\right)\leq Z_{max},$$

where $Z_{min}$ and $Z_{max}$ denote the minimum and maximum atomic numbers among the constituent elements.^14^

Midgley demonstrated that, depending on the range of x‐ray energies and material compositions considered, fewer coefficients may be sufficient. However, for elements across the entire periodic table and at energies above their K‐edge and up to 125 MeV, using N=5 coefficients reproduce the tabulated elemental cross‐sections with an absolute difference of less than 2%,^14^ providing robust coverage across a broad energy spectrum and variety of materials.

**FIGURE 1:**
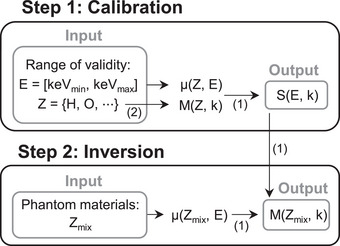
Schematic overview of the steps involved in the material analysis process using Midgley's parameterization: step 1 (top) determines the energy‐dependent coefficients for a fixed energy and compositional range of interest; step 2 (bottom) calculates the mixture parameters for all materials in the phantom that fall within the range of validity of the parameterization established in step 1. Numbers in parentheses refer to the corresponding equations in the text.

For material analysis, the implementation of this parameterization involves two steps, as shown in Figure 1. The first step determines the coefficients S(E,k) over the selected energy and compositional ranges of validity of the parameterization for the intended application. These coefficients are obtained by solving a least‐squares problem using attenuation coefficients μ(Z,E) and mixture parameters M(Z,k) across the chosen range of x‐ray energies and reference elements. Then, the inversion step retrieves the unknown mixture parameters of a material by solving a least‐squares problem using its measured attenuation coefficients $\mu (Z_{mix},E)$ and the previously computed S(E,k). Attenuation data were sourced from x‐ray DB,^16^, ^17^ using elemental compositions reported in prior studies.^18^, ^19^, ^20^, ^21^

Therefore, this parameterization allows for the representation of any material within the valid energy and composition range using a finite number (N) of mixture parameters. These coefficients are then used for phantom representation. Specifically, unlike the conventional phantom representation, where each voxel is assigned a material index linked to known attenuation properties, the proposed approach replaces these indices with the composition‐dependent parameters. The phantom is thus expanded into N channels, each encoding one parameter, so that each voxel is represented by N values instead of a single index.

### 2.2 Simulation and reconstruction framework

In conventional deterministic simulations using ray‐tracing,^22^ projection data is generated based on the discretized Lambert‐Beer law:

(5) $$\vec{y}=\underset{E}{\sum }{\vec{b}}_{E}\exp\left(-L\cdot \vec{\mu }\left(E\right)\right)\;$$

Here, ${\vec{b}}_{E}$ represents the parameter combining the x‐ray spectrum and detector energy response, L is the projection matrix, and $\vec{\mu }\left(E\right)=\vec{\phi }\left(E\right)\times \rho$ is the linear attenuation coefficient at energy E, where $\vec{\phi }\left(E\right)$ is the mass attenuation coefficient and ρ is the mass density.

Line integrals are computed by tracing rays through the voxelized volume. When a ray intersects voxel boundaries, interpolation is applied, and partial volume effects may occur due to the transition between materials along the ray path.^23^ The resulting projection data are unitless sinograms that represent the fraction of the transmitted signal.

**FIGURE 2:**
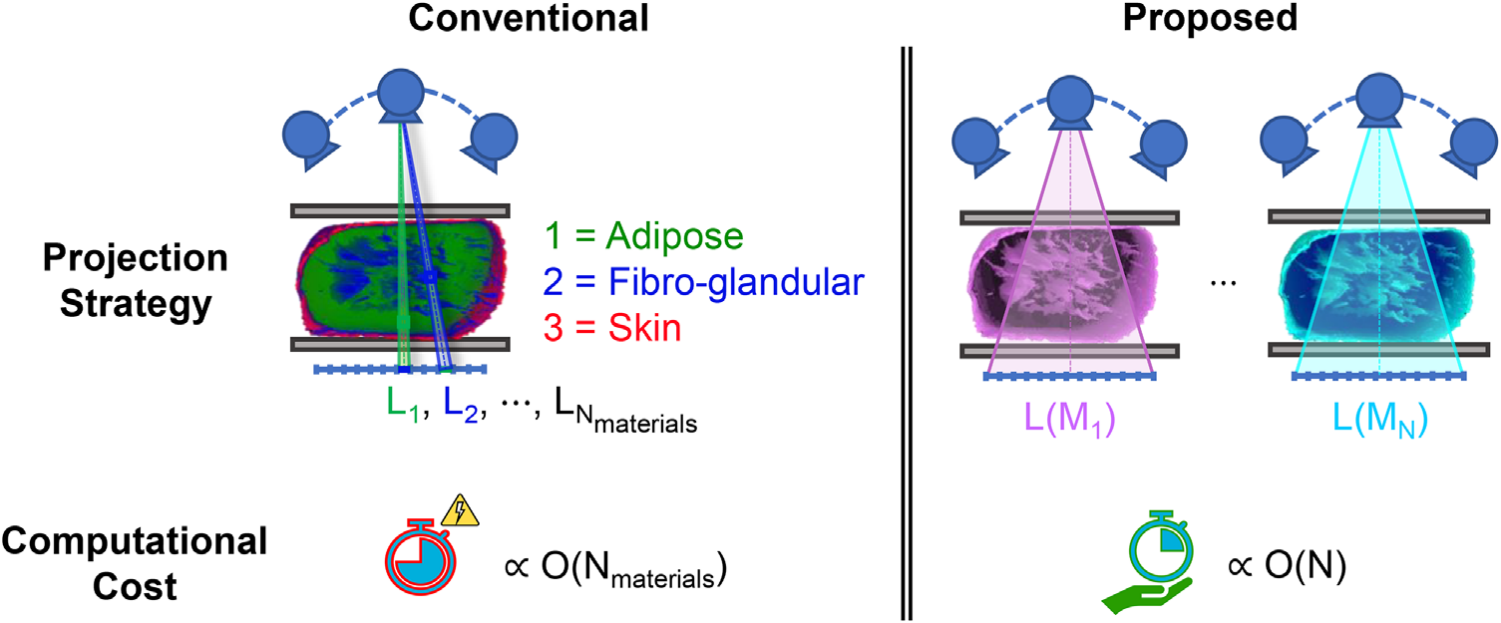
Illustrative comparison between the conventional and proposed methods. Left: The conventional approach assigns a unique index to each tissue type (i.e., adipose, fibro‐glandular, and skin) and performs individual projections for each (top), resulting in computation time that increases with the number of materials and compositional variations within the phantom (bottom). Right: The proposed method uses Midgley's parameterization, representing all materials with *N* = 5 parameters and requiring only *N* forward projections regardless of material complexity (top), resulting in a fixed computational cost (bottom).

This simulation approach becomes computationally expensive as material complexity increases. Therefore, we propose a parameterized simulation method. Starting from the phantom representation detailed in Section 2.1, the proposed method forward‐projects the N mixture parameter channels, which are then scaled by the corresponding S‐curves. As a result, the number of forward projections is fixed at N, regardless of the number of materials and compositional variations involved. Figure 2 provides a side‐by‐side comparison of phantom representation, projection strategy, and computational cost for the conventional and proposed methods, using a compressed breast phantom as an example.

Algorithm 1 compares the two simulation strategies: the conventional approach iterates over material indices, while the parameterized method iterates over the reduced set of mixture parameters.

ALGORITHM 1 **Pseudo‐code describing the key steps of the simulation algorithms. In conventional simulations, voxels associated with compositional variations ($V_{i}$) are individually projected and scaled by their corresponding attenuation coefficients (${\mu_{i}}^{\left(E\right)}$). The proposed parameterization, instead, projects the volume associated with each channel ($V_{c}$) and scales it by the S‐parameters (${S_{c}}^{\left(E\right)}$).**

**Table:** 

**Algorithm** **1**	
**Conventional Simulation**	**Proposed Simulation**
**Require**: $\mu_{i}^{\left(E\right)}$, $b_{E}$, $V_{i}$	**Require**: $S_{c}^{\left(E\right)}$, $b_{E}$, $V_{c}$
1: **for all** indexes i **do**	1: **for all** channels c **do**
2: $L_{i}\leftarrow proj\left(V_{i}\right)$	2: $L_{c}\leftarrow proj\left(V_{c}\right)$
3: **end for**	3: **end for**
4: **for all** energy bins E **do**	4: **for all** energy bins E **do**
5: $worksino\leftarrow \sum_{i}\mu_{i}^{\left(E\right)}\cdot L_{i}$	5: $worksino\leftarrow \sum_{c}S_{c}^{\left(E\right)}\cdot L_{c}$
6: $worksino\leftarrow b_{E}\cdot \exp\left(-worksino\right)$	6: $worksino\leftarrow b_{E}\cdot \exp\left(-worksino\right)$
7: sino←sino+worksino	7: sino←sino+worksino
8: **end for**	8: **end for**

Sinograms from both simulation methods were then reconstructed using the maximum likelihood for transmission method^24^ with 100 iterations, a reconstruction method independent of any material parameterization model and therefore free from inverse crime concerns in that regard.

### 2.3 Applications

The proposed simulation approach was evaluated in two scenarios addressing different sources of anatomical complexity: modeling tissue variability in breast imaging and simulating a multitude of anatomical structures in whole‐body imaging.

The breast imaging use case focuses on the framework's ability to accurately simulate tissue variability under idealized conditions, allowing a focused assessment of variability modeling without the influence of external factors, while the whole‐body CT imaging use case shifts focus to realistic anatomical representation and imaging conditions.

#### 2.3.1 Tissue variability in breast imaging

As a first use case, we evaluated the proposed framework in simulating fibro‐glandular tissue variability in DBT. The same approach can be extended to other breast tissues and imaging modalities.

##### Dataset

The dataset consisted of 10 digital compressed breast phantoms with a voxel size of 0.273 mm and thickness ranging from 30 to 82 mm, composed of skin, adipose, and fibro‐glandular tissue.^25^ Tissue elemental compositions were taken from prior tabulations,^18^ and converted to attenuation coefficients.^16^

**FIGURE 3:**
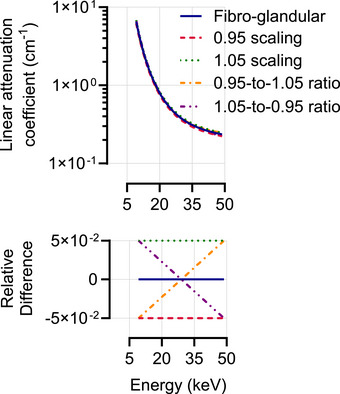
Top: Nominal fibro‐glandular attenuation curve (dark blue) along with its possible variations: less dense (dark red), denser (dark green), less steep (orange), and steeper (purple) curves. Bottom: Relative difference between the nominal curve and each of its variations.

To introduce variability in the fibro‐glandular tissue, each voxel was assigned a variation of the fibro‐glandular attenuation curve. Variations were modeled from the nominal attenuation curve by defining four extremes based on combinations of offset and slope: the offset was shifted by ± 5% to represent tissue density variations,^6^ and the slopes were set so that one curve spanned from the left extreme of the –5% offset to the right extreme of the +5% offset (shallowest), while the other spanned the opposite extremes (steepest) (Figure 3). All remaining curves were interpolated within these extremes. In this study, a total of 200 different attenuation curves were generated and randomly assigned to fibro‐glandular voxels, resulting in 203 distinct tissue variations per phantom, including the three normal tissues.

Midgley's parameterization was applied to both the original phantoms and those modified to include tissue variability, generating five mixture parameters each. The parameterization was defined over an energy range of 9 to 49 keV, with the lower bound set above the filtration cutoff and the K‐edges of the relevant elements, and the upper bound selected to cover the diagnostic range relevant to DBT. The compositional range was selected as representative of breast tissue, including elements H, C, N, O, Na, P, S, Cl, K, and Ca, based on published tissue analyses.^18^

This process resulted in two representations for both the original breast phantoms and those including fibro‐glandular tissue variability: the representation indexed by tissue type, and the one expressed using Midgley's mixture parameters, as described in Section 2.1.

##### Image simulations and reconstructions

Projection data were simulated using the geometry corresponding to a clinical DBT system (MAMMOMAT Revelation, Siemens Healthineers, Erlangen, Germany). The x‐ray source rotated over a ± 25° arc relative to a stationary detector, acquiring 25 uniformly spaced projections. System geometry included a source‐to‐isocenter distance of 60.85 cm, a source‐to‐detector distance of 65.55 cm, and a phantom position offset of 3.3 cm below the isocenter. The tungsten spectrum was adjusted using automatic exposure control based on breast thickness, with 50 µm rhodium filtration and normalization applied. The detector size was 30 × 24 cm^2^, with a pixel pitch of 85 µm.

Images were simulated and reconstructed as described in Section 2.2, using both the conventional and proposed simulation methods. Simulated projection data excluded factors such as resolution loss, noise, or x‐ray scatter to allow a focused assessment of variability modeling without the influence of external factors.

#### 2.3.2 Anatomical variability in whole‐body imaging

As a second use case, we applied the proposed framework to simulate whole‐body CT scans using XCAT phantoms, which provide detailed anatomical structures composed of many different materials.

##### Dataset

The dataset consisted of two XCAT phantoms^1^ with a voxel size of 0.25 mm, one representing the head and the other the abdomen. The head phantom was composed of 9 materials: adipose tissue, skeletal muscle tissue, cortical bone, blood, cartilage, cerebrospinal fluid, white and grey matter, and air. The abdomen phantom was composed of 13 materials: on top of the first four materials present in the head, spongiosa, lung parenchyma, pancreas, gallbladder, abdomen, kidney, spleen, skin, and water. Material elemental compositions were taken from prior tabulations,^19^, ^20^, ^21^ and converted to attenuation coefficients.^16^

Midgley's parameterization was applied to these phantoms, generating five mixture parameters each. The parameterization used an energy range of 22 to 80 keV for the head, and 27 to 120 keV for the abdomen phantoms, with lower bounds set above the filtration cutoff and the K‐edges of the relevant elements, and upper bounds selected to cover the diagnostic energy ranges for CT. The compositional range included the same elements as for the breast phantoms with the addition of Mg and Fe, based on published analyses of head and abdomen materials.^19^, ^20^, ^21^

Again, this process resulted in two representations for both the head and abdomen phantoms: the original representation indexed by material type, and the one expressed using Midgley's mixture parameters.

##### Image simulations and reconstructions

Projection data were simulated using a geometry corresponding to a 320‐row CT system (Aquilion ONE PRISM Edition, Canon Medical Systems, Otawara, Japan). The specific geometry parameters of this clinical CT scanner are vendor‐specific and confidential. X‐ray spectra were set to 80 kV for the head and 120 kV for the abdomen phantom, with bowtie filtration and normalization applied.

Images were simulated and reconstructed as described in Section 2.2, using both the conventional and proposed simulation methods. To reflect realistic acquisition conditions, system‐specific spatial resolution and noise characteristics were introduced through post‐processing of the simulated sinograms, following previously developed image modification routines.^26^, ^27^ Spatial resolution was modeled by applying the pre‐sampled modulation transfer function (MTF) to the super‐sampled sinogram. Noise was added to the resolution‐adjusted sinogram using the normalized noise power spectrum (NPS) and scaled according to the mean‐variance relationship of the noise signal. The MTF, NPS, and mean‐variance relationship were modeled from prior characterizations of the same scanner.^27^ Since the material representation affects only the projection stage, these image modification routines were applied identically to both the conventional and proposed simulation strategies without altering downstream processing.

Image reconstruction was performed using a volume size of 512 × 512 × 320 voxels. The reconstruction field of view was set to 240 × 240 × 160 mm^3^ for the head and 400 × 400 × 160 mm^3^ for the abdomen.

### 2.4 Analysis

To assess the accuracy of the proposed framework, the percent error between results from the proposed parameterized simulation method and those from the conventional non‐parameterized method was used as the primary evaluation metric. Additionally, the root mean squared error (RMSE) was computed as a complementary metric to provide an absolute measure of error and aid the interpretation of results.

The analysis started with a validation of Midgley's parameterization by comparing predicted linear attenuation coefficients, calculated as a linear combination of S‐curves and mixture parameters according to equation (1), to nominal values derived from the mixture rule. For reference, standard two‐material models such as Al/PMMA and bone/water were also included. To ensure fair comparison and prevent bias, bone and water were excluded from error calculations when present in the phantom, since their exact match would lower overall error estimates.

In the projection and reconstruction domains, accuracy was assessed by computing percent errors between sinograms and reconstructed images generated using the two simulation methods, with results summarized by the median and 5th‐95th percentile values to limit the influence of outliers. For context, the projection‐domain performance was also compared to standard two‐material models; in these comparisons, cortical bone and water were replaced with mineral bone and soft tissue to avoid bias across models.

The spatial distribution of errors was examined using pixel‐wise heat maps for the projections with the highest overall error, displaying both absolute difference and absolute percent error maps.

When relevant to the experimental design, extra analyses were performed. In the breast application with voxel‐wise variability, where photon energies fall outside the range where fewer than five coefficients may be sufficient, the effect of the number of Midgley coefficients was evaluated by repeating simulations with N=2,3,4, and 5. In the whole‐body imaging application, where complexity increases, three further analyses were conducted. First, to place the observed errors in the context of standard simulation variability, since no absolute ground truth exists, results from the proposed method were compared to differences between ideal conventional simulations using two different projector models: a distance‐driven projection approach,^28^ which weighs voxel contributions by ray‐voxel intersection areas, and the pixel‐driven ray‐tracing method used throughout this study,^22^ in which rays are traced from each detector pixel through the voxelized volume. Second, to assess robustness under realistic imaging conditions, the percent error analysis was repeated after applying spatial resolution loss and noise modeling routines to both simulation methods.

Third, computational efficiency was evaluated by comparing simulation times for the two methods using a Wilcoxon signed‐rank test over 10 repeated runs. To ensure a fair comparison, the time required for the parameterization process, including the phantom conversion to the parameterized representation, was also evaluated. Observed time reductions were examined relative to the ratio of the number of materials used in each simulation to verify that the acceleration scaled linearly with the number of materials present in the phantoms, as theoretically expected. All simulations were executed on a workstation with an Intel Xeon Silver 4310 processor (2.1 GHz, 48 cores), 512 GB of RAM, and an NVIDIA A100 GPU with 80 GB of memory. Computations were GPU‐accelerated using CUDA (v12.5), with projections processed in memory‐efficient batches to avoid exceeding GPU memory.

In all analyses, open‐field regions were excluded. These were identified in the projection domain using known simulated open‐field values and, in the reconstruction domain, via Otsu's thresholding with manual refinement to separate materials from the open field.

## 3 RESULTS

### 3.1 Midgley's parameterization

**FIGURE 4:**
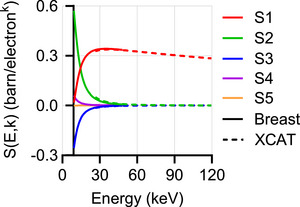
S‐curves computed by solving a least‐square problem within the specified energy and compositional range of interest for both breast (solid line) and XCAT phantoms (dashed line).

Figure 4 shows the S‐parameters across the specified energy and compositional ranges of interest for both breast and XCAT phantoms.

Table 1 reports the median and interquartile (IQR) range of the percent error between attenuation coefficients obtained through the proposed least‐squares fitting approach and nominal values derived from the mixture rule, and compares it to those from other commonly used parameterization models.

**TABLE 1:** Median and IQR range (in %) of the error between linear attenuation coefficients obtained using each of the three parameterization models and the nominal values, computed across all energy bins and materials present in each phantom. Bone and water materials were excluded from the error calculation when present in the phantom.

Median (IQR) error (%)	Breast	Head	Abdomen
Proposed	0.20 (−1.18; 1.75) × 10^−3^	0.11 (−1.54; 1.57) × 10^−3^	−0.17 (−1.21; 1.28) × 10^−3^
Al/PMMA	0.24. (−2.37; 2.39) × 10^−1^	0.01 (−1.25; 2.16) × 10^−2^	−0.88 (−3.18; 7.21) × 10^−3^
Bone/water	−3.06 (−8.29; 3.24) × 10^−1^	−0.13 (−3.45; 2.28) × 10^−2^	−0.04 (−1.55; 0.66) × 10^−2^

**FIGURE 5:**
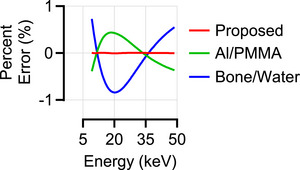
Percent error (%) of the skin tissue attenuation coefficient, calculated relative to the mixture rule values, for the proposed parameterization (red), Al/PMMA (green), and bone/water (blue) models.

Among all phantoms, breast tissues showed the highest maximum absolute percent error: 0.007% for the breast, compared to 0.004% for the head and 0.003% for the abdomen. Figure 5 shows the percent error for skin tissue, which had the highest error among the breast tissues, alongside results from the other parameterization models.

These percent errors correspond to RMSE values across all energy bins and materials of 3.24 × 10^−5^ cm^−1^, 0.54 × 10^−5^ cm^−1^, and 0.40 × 10^−5^ cm^−1^ for breast, head, and abdomen phantoms, respectively.

### 3.2 Projection domain

**FIGURE 6:**
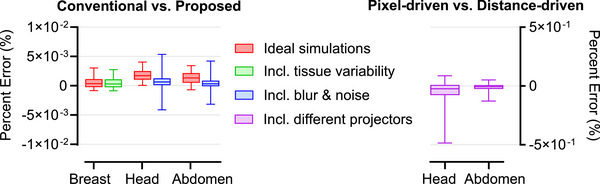
Left: Boxplots of percent errors (in %) between sinograms generated by the conventional and proposed simulation methods. The horizontal line within each box indicates the median, and the whiskers the 5^th^‐95^th^ percentiles. For breast phantoms, errors are shown without (red) and with (green) tissue variability. For XCAT phantoms, errors are reported for ideal sinograms (red) and after applying image modification routines (blue). Right: For reference, ideal sinogram errors from two projector models (pixel‐driven vs. distance‐driven) are shown for the XCAT phantoms to contextualize the accuracy of the proposed method to typical simulation variability in ideal scenarios.

Figure 6 summarizes the accuracy of the proposed framework, reporting the median and 5th–95th percentiles of percent errors between sinograms from the conventional and proposed methods. These errors were computed across all sinogram pixels and, for the breast case, across all phantoms. Breast imaging results are shown both with and without fibro‐glandular tissue variability, while whole‐body results include both ideal and realistic simulations incorporating resolution loss and noise modeling. For reference, errors from ideal conventional simulations using an alternative projector model are also shown to illustrate standard simulation variability in ideal scenarios.

Across all pixels, the RMSE computed under controlled conditions (without tissue variability for the breast and using ideal scenarios for the XCAT phantoms) was 1.42 × 10^−6^ for the breast, 0.56 × 10^−6^ for the head, and 0.41 × 10^−6^ for the abdomen.

**FIGURE 7:**
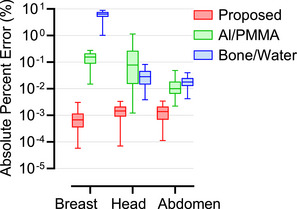
Boxplots of absolute percent errors (in %), on a logarithmic scale, between sinograms generated by the conventional non‐parameterized simulation method and those generated using three parameterization approaches: the proposed method (red), Al/PMMA (green), and bone/water (blue). The horizontal line within each box indicates the median, and the whiskers the 5th–95th percentiles. Breast errors are shown without tissue variability, while XCAT errors correspond to ideal simulation conditions.

Figure 7 compares the proposed parameterization to other parameterization models in the projection domain, reporting median and 5th–95th percentile absolute percent errors between conventional and parameterized simulations for all phantoms under controlled conditions.

**FIGURE 8:**
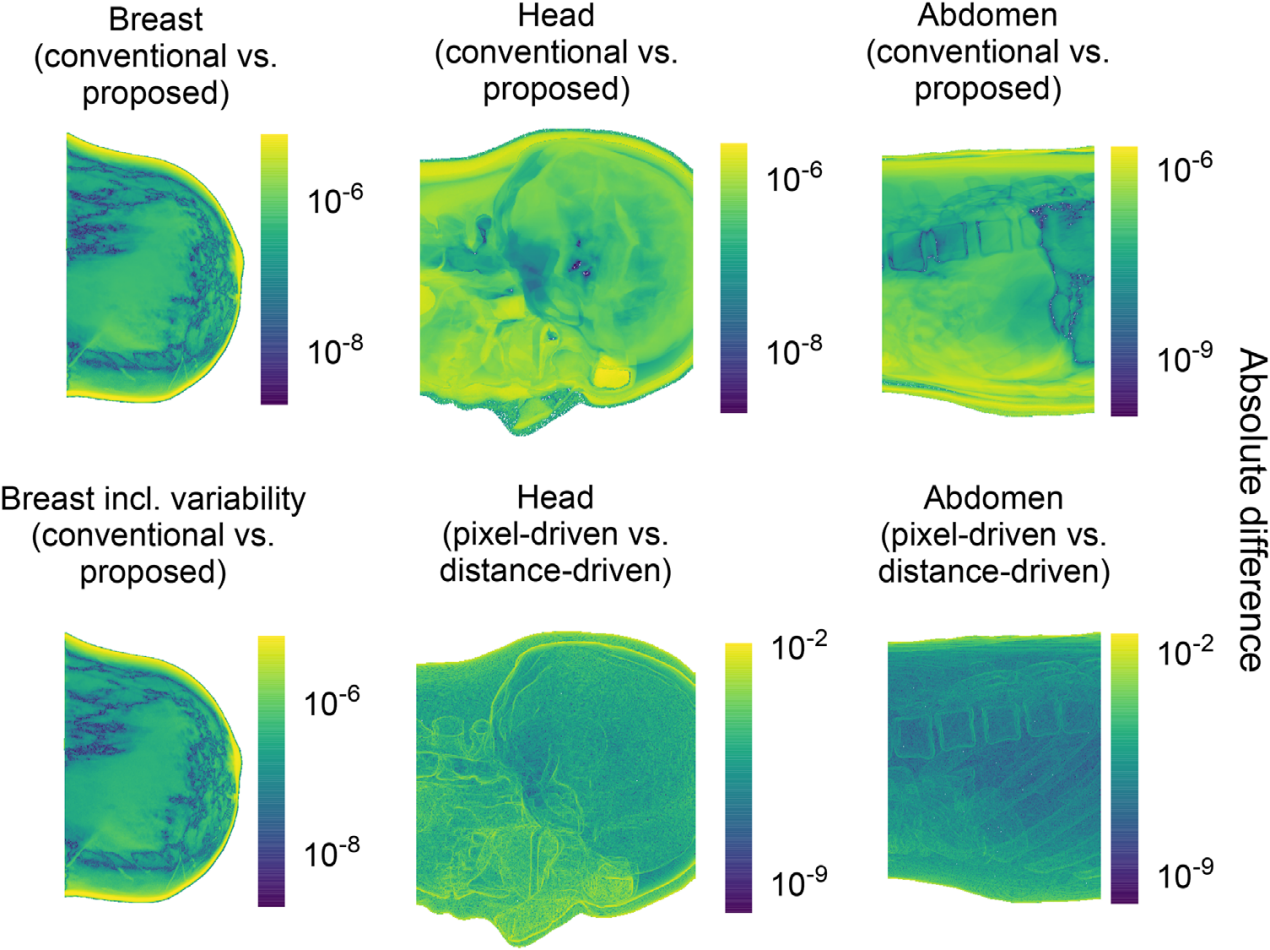
Pixel‐wise absolute difference heat maps, on a logarithmic scale, comparing sinograms from the ideal conventional and proposed simulation methods. For each phantom type, the projection with the highest overall difference is shown. Left column: breast phantom without (top) and with (bottom) tissue variability. Middle column: head phantom (top), compared to the difference between pixel‐driven and distance‐driven projectors using ideal conventional simulations (bottom). Right column: abdomen phantom (top), compared to the difference between pixel‐driven and distance‐driven projectors (bottom).

**FIGURE 9:**
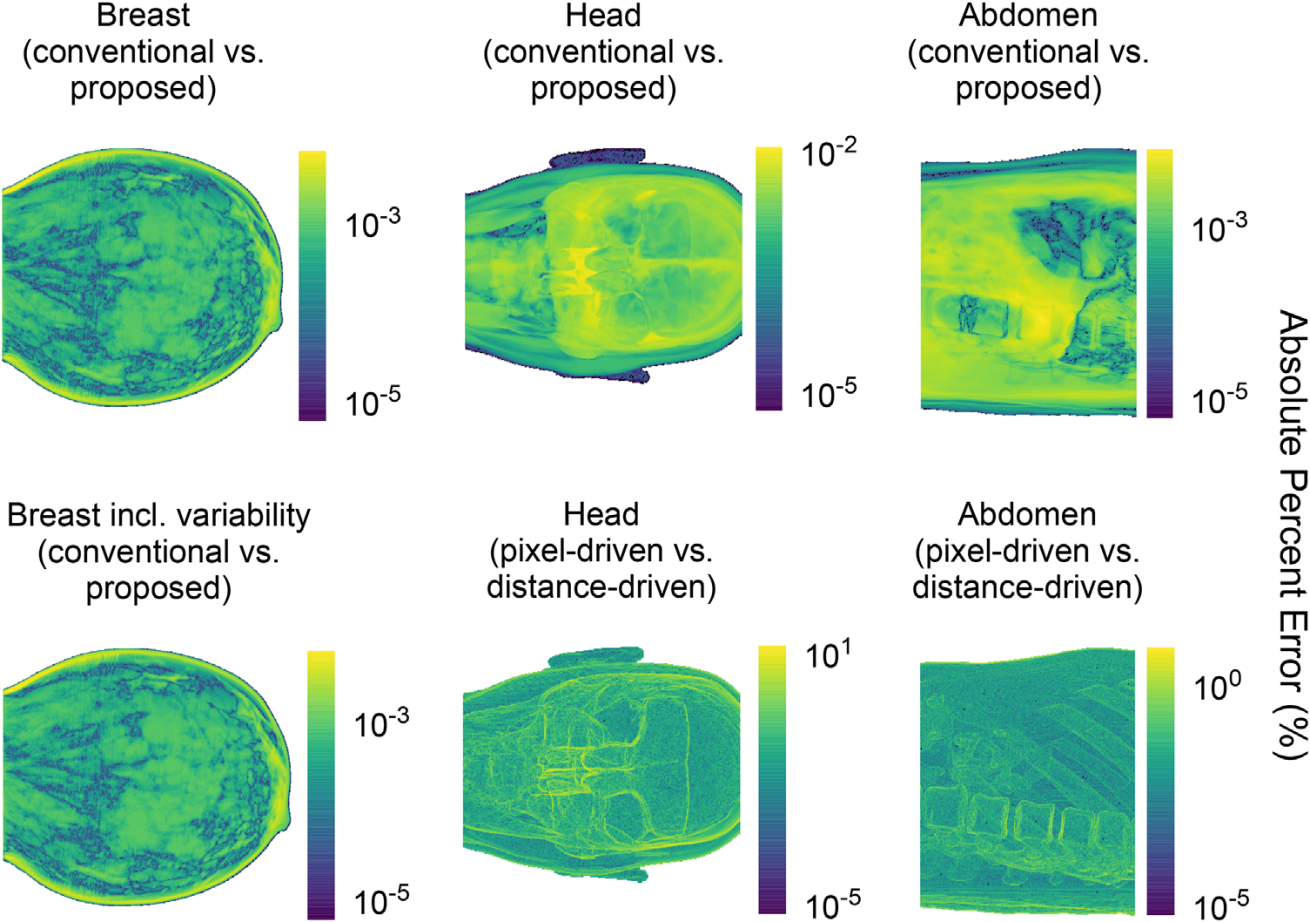
Pixel‐wise absolute percent error heat maps (in %), on a logarithmic scale, comparing sinograms from the ideal conventional and proposed simulation methods. For each phantom type, the projection with the highest overall error is shown. Left column: breast phantom without (top) and with (bottom) tissue variability. Middle column: head phantom (top), compared to the error between pixel‐driven and distance‐driven projectors using ideal conventional simulations (bottom). Right column: abdomen phantom (top), compared to the error between pixel‐driven and distance‐driven projectors (bottom).

Figures 8 and 9 display heat maps of absolute differences and absolute percent errors, respectively, for the projection with the highest error for each phantom. For the XCAT phantoms, additional heat maps using an alternative projector model provide a benchmark for expected variability. Errors slightly increased near high‐contrast boundaries, such as air–tissue interfaces.

**FIGURE 10:**
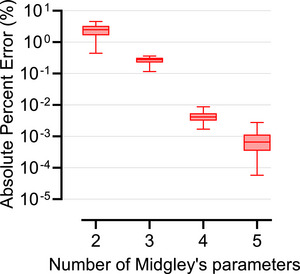
Boxplots of absolute percent errors (in %), on a logarithmic scale, between sinograms from the conventional non‐parameterized simulation and the proposed parameterized simulation using 2 to 5 Midgley coefficients for the breast phantoms with tissue variability. The horizontal line within each box indicates the median, and the whiskers the 5th–95th percentiles.

Finally, Figure 10 shows the effect of the number of Midgley coefficients. Using N=2 or 3 resulted in substantially higher errors, N=4 improved accuracy, and N=5 reduced median error to below 10^−^^3^ across all sinograms.

### 3.3 Reconstruction domain

**FIGURE 11:**
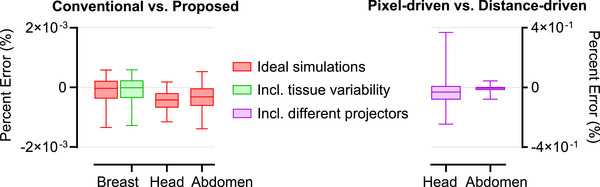
Left: Boxplots of percent errors (in %) between reconstructed images obtained using the conventional and proposed methods. The horizontal line within each box indicates the median, and the whiskers the 5th–95th percentiles. For breast phantoms, errors are shown without (red) and with (green) tissue variability. For XCAT phantoms, errors are reported for ideal scenarios (red). Right: For reference, reconstruction errors from different projector models (pixel‐driven vs. distance‐driven) are shown for the XCAT phantoms to contextualize the accuracy of the proposed method to typical simulation variability in ideal scenarios.

Figure 11 shows the percent error between reconstructed images from the conventional and proposed methods, summarized by the median and 5th–95th percentile values. Errors were computed over all reconstructed voxels and, in the breast imaging case, across all phantoms. Breast imaging results are reported both with and without fibro‐glandular tissue variability, while whole‐body imaging results also include reference errors from ideal conventional simulations using alternative projector models. The maximum voxel‐wise errors were 0.005% in the breast phantoms, 0.010% in the head phantom, and 0.005% in the abdomen phantom.

### 3.4 Computational time

The proposed method significantly reduced computation time compared with the conventional approach. For the head phantom, the mean ± standard deviation over 10 repeated runs was 10.90 ± 0.06 s for the proposed method versus 19.81 ± 0.24 s for the conventional method (*p* < 0.01). For the abdomen phantom, the proposed method required 20.94 ± 0.03 s compared with 54.05 ± 0.31 s for the conventional approach (*p* < 0.01). The parameterization process, including the conversion of digital phantoms into the proposed representation, was also computationally efficient, requiring only 0.82 ± 0.42 s for the head phantom and 1.61 ± 0.18 s for the abdomen phantom.

The observed acceleration factors were 1.8 for the head and 2.6 for the abdomen phantom, consistent with the expected ratios derived from the number of materials used in each simulation (9/5 = 1.8 for head; 13/5 = 2.6 for abdomen).

## 4 DISCUSSION

The development of efficient and realistic simulation frameworks is essential for advancing imaging system design, optimization, and validation. This study integrated Midgley's parameterization into a deterministic simulation pipeline and demonstrated that it can accurately model material attenuation while reducing computational time in anatomically detailed scenarios.

The parameterization model showed excellent agreement with nominal attenuation values, outperforming commonly used two‐material parameterization models in both accuracy and robustness across different anatomical regions. The maximum absolute percent errors in linear attenuation coefficients were 0.007% for breast phantoms and 0.004% for XCAT phantoms, corresponding to absolute deviations of 3.24 × 10^−5^ cm^−1^ and 0.54 × 10^−5^ cm^−1^, respectively, which are well below reported biological and scanner‐related variability. For reference, previous studies have reported a measured variability in breast tissue attenuation of approximately 4%,^6^ while inter‐scanner variability in whole‐body soft‐tissue Hounsfield Units is typically around 10%, with the liver reaching deviations of about 30%.^29^ These results confirm that Midgley's representation can reliably parameterize material attenuation properties within realistic variability bounds.

Projection‐domain comparisons between the proposed and conventional simulations (Figure 6) showed errors within ± 0.006% across all phantoms, corresponding to absolute deviations on the order of 10^−6^, demonstrating the high accuracy of the proposed simulation framework. These values were smaller than the errors observed when switching between projector models (± 0.5%), which served as a benchmark for typical simulation variability in ideal scenarios. This suggests that the parameterization introduces negligible deviations compared to the intrinsic uncertainties of standard simulation pipelines. High accuracy was preserved across both DBT and whole‐body CT applications, highlighting the generalizability of the framework across different anatomical regions and imaging modalities. Moreover, incorporating realistic resolution loss reduced high‐frequency discrepancies, resulting in a more concentrated error distribution around zero, while noise modeling introduced random pixel‐level fluctuations.

Comparisons with conventional two‐material parameterizations in the projection domain revealed larger errors when the basis materials did not match the tissue composition, as for the bone/water parameterization in the breast (Figure 7). In contrast, Midgley's parameterization consistently produced lower errors across all anatomical regions and energy ranges, demonstrating its robustness and generalizability.

Regarding the spatial distribution of errors, the highest errors were localized at high‐contrast interfaces, particularly in the nasal region of the head phantom, where sharp air‐tissue gradients challenge ray‐tracing simulations due to non‐linear partial volume effects (Figure 9). However, such effects are intrinsic to the simulation method and independent of phantom parameterization.

The impact of the number of Midgley coefficients was also evaluated (Figure 10). While four coefficients achieved high accuracy, using five further reduced errors and captured subtle effects, justifying the choice of five coefficients for robust parameterization. This aligns with Midgley's findings, which indicate that four coefficients are sufficient only within limited energy and compositional ranges, with additional coefficients needed when these ranges are exceeded.

Reconstruction‐domain analyses (Figure 11) followed the projection‐domain trends, confirming the accuracy and robustness of the proposed framework throughout the imaging pipeline.

From a computational perspective, the proposed framework offers a clear advantage over conventional material indexing methods, where simulation time scales with the number of material compositional variations. The proposed approach maintains a fixed cost equivalent to five projections, regardless of anatomical complexity, resulting in significant time savings for the XCAT phantoms. Acceleration factors were 1.8 for the head and 2.6 for the abdomen, in line with theoretical expectations based on material ratios, confirming that computational time scales linearly with the number of phantom materials. The parameterization step adds minimal overhead, making the method particularly suitable for applications requiring high anatomical realism or extensive material heterogeneity.

By avoiding the use of common two‐material parameterization models, the proposed method is particularly useful for spectral imaging studies, where these models are often used for material decomposition, and their inclusion in the simulation pipeline would introduce systematic biases and result in the inverse crime. In addition, beyond the scenarios evaluated in this study, Midgley's parameterization allows continuous, voxel‐level modulation of attenuation coefficients within physically constrained bounds. This feature can be used to model material variability and gradual transitions at material interfaces, while ensuring realistic material properties, making it a valuable tool for future simulation studies involving high‐fidelity phantoms and partial‐volume effects.

This parameterization approach is inherently tied to deterministic projection‐based simulations and cannot be directly applied to MC frameworks for modeling stochastic photon interactions, such as scattering or fluorescent emission. However, its demonstrated accuracy, robustness, and computational efficiency make it a powerful tool for a wide range of imaging research applications where computational resources are limited and stochastic modeling is not essential.

## 5 CONCLUSIONS

This study demonstrates that Midgley's parameterization provides an accurate and computationally efficient simulation framework for modeling material attenuation in anatomically realistic x‐ray imaging. By representing complex mixtures with a compact set of parameters, the proposed approach overcomes the computational challenges posed by detailed anatomical structures and variability. Validated on both breast and whole‐body phantoms, this framework offers a promising alternative to conventional simulation techniques, supporting the development and optimization of x‐ray imaging systems, especially in spectral imaging applications.

## CONFLICT OF INTEREST STATEMENT

Ioannis Sechopoulos has research agreements with Siemens Healthcare, Canon Medical Systems, ScreenPoint Medical, Sectra Benelux, Volpara Healthcare, Lunit, a speaker agreement with Siemens Healthcare, and is a Scientific Advisory Board member of Koning Corp.

## Supporting information




Supporting information
